# The impact of hydroclimate-driven periodic runoff on hydropower production and management

**DOI:** 10.1038/s41598-024-76461-3

**Published:** 2024-10-29

**Authors:** Shuang Hao, Anders Wörman, Luigia Brandimarte

**Affiliations:** Department of Sustainable Development, Environmental Science and Engineering (SEED), KTH Royal Institute of Technology, Stockholm, 100 44 Sweden

**Keywords:** Biennial periodicity, Stochastic forecasting, Scenarios, Optimisation of hydropower, Wet-year, Dry-year, Hydroelectricity, Hydrology

## Abstract

This study evaluates the impact of hydroclimate-driven periodic runoff on hydropower operations and production, with a focus on how the forecasted biennial periodicity of runoff time series could affect the efficiency of hydropower generation. Hydrologic stochastic processes are utilized to forecast long-term runoff, and seven hydroclimate scenarios are developed to be input into a production management model, allowing for an analysis of how periodic hydroclimate variations influence hydropower management and output. The results reveal that the biennial alternation between wet and dry years is a key factor affecting hydropower operations in the Dalälven River Basin. Notable differences between wet- and dry-year scenarios were observed in terms of power efficiency, production output, and forecasting accuracy. Operating hydropower systems based on dry-year runoff forecasts in wet years results in a 1.63% decrease in production efficiency and a reduction of 9,104 MWh in power generation. Conversely, applying wet-year forecasts in dry years slightly boosts production efficiency by 0.31% and increases power generation by 7,832 MWh. Scenarios that adhere to biennial periodicity offer the highest forecasting accuracy, particularly when applying dry-year forecasts in dry years in winter and spring, which produce the most precise predictions. In contrast, using dry-year forecasts in wet years results in the lowest forecasting accuracy.

**Supplementary Information:**

The online version contains supplementary material available at 10.1038/s41598-024-76461-3.

## Introduction

Hydropower contributes 55% of global renewable electricity production and provides nearly 30% of the world’s flexible electricity supply^1^. In countries like Nepal and Norway, hydropower accounts for nearly 90% of the total electricity production^2–4^. Despite its critical role in the energy market, hydropower production is influenced by various hydroclimate periodicities, including seasonal, yearly and biennial cycles^5–7^. These periodic fluctuations can increase variability in streamflow, reduce the accuracy of hydroclimate forecasts, and complicate the planning and management of hydropower production.

Several studies have examined periodicity in hydrology and hydroclimate. Lovino et al. (2018) identified interannual hydroclimatic variability centred around 2.5–6.5 years, corresponding to El Niño Southern Oscillation (ENSO) periodicities. Uvo et al. (2021) observed climate-driven streamflow variability in oscillation bands of 2–4 years and 8–16 years. Additionally, climate-driven fluctuations in power of runoff were noted in annual, biennial and 8-year cycles^8^. Biennial periodicity, in particular, has been documented across numerous studies. For instance, periodic variations in water availability, ranging from 2.1 to 2.5 years, were found in 18 drainage basins^9^. Temperature and precipitation oscillations of approximately 2–3 years were detected in Siberia and East Asia using the maximum entropy method and Fourier spectral analysis^10^. However, the connection between biennial hydrological periodicity and hydropower production remains unclear and open to further investigation.

Most studies analysing the impact of climate change on hydropower focus primarily on climate-related factors such as CO2 emission, temperature changes and other atmospheric effects. These studies often use Global and Regional Climate Models to examine various emission scenarios^11–16^ across different spatial scales. However, only a limited number of studies have explored the effects of hydroclimate periodicity on hydropower production, leaving a gap in understanding how these periodic fluctuations influence energy output.

Capturing the role of hydroclimate periodicity is essential for improving the planning and management of hydropower systems. This paper addresses this gap by assessing the significance and impact of forecasted biennial periodicity on hydropower production and management. The key innovations of this study are as follows: (a) hydrologic stochastic forecasting is employed to account for long-term hydroclimate periodicity, reflecting the nonlinear and statistical nature of hydrology processes; (b) seven hydroclimate scenarios are developed to explore the matching and mismatching periodic climate modes on hydropower production; (c) the study adopts a long-term assessment approach, focusing on time frames of three months and half a year, offering valuable insights for long-term hydropower management.

A major contribution of this study is its comprehensive assessment method, which integrates hydrologic stochastic forecasting, a cascade hydropower production and management model, Monte Carlo simulations, forecasting performance evaluations, and its application to a real-world case in the Dalälven River Basin.

The proposed assessment process has been implemented in a MATLAB environment and simulated on a high-performance computer platform, supported by National Academic Infrastructure for Supercomputing in Sweden (NAISS), which significantly enhances computational efficiency.

The structure of the paper is as follows. Section 2 describes the methodology, detailing the stochastic forecasting processes, forecasting performance metrics, hydropower production model, assessment model, Monte Carlo simulations, and biennial periodicity scenarios. Section 3 describes the case study area and data sources. Section 4 presents the simulation results and discusses the implications. Finally, Sect. 5 offers conclusions regarding the impact of hydroclimate periodicity on hydropower production.

## Methodology

The primary aim of this research is to investigate whether, and how, hydroclimate with biennial periodicity can improve hydropower production in the long term (3 months and 6 months). To achieve this, the study employs a range of methods, including stochastic forecasting, performance evaluation of forecasts, hydropower production simulation, an impact assessment model, Monte Carlo simulations, and the development of biennial forecasting scenarios.

### Hydrologic stochastic processes for forecasting

Various methods in stochastic hydrology can be used to model the random processes inherent in hydrology, such as the combination of erratic and systematic climate variations^17,18^ considered in this study. Information on the hydrologic stochastic processes can be extracted from historical data and expressed as segmented time-series samples, which are then used in forecasting^19^. In this study, time series from wet and dry years are segmented, and specific segments are randomly extracted for forecasting.

The rationale for using hydrologic stochastic processes, based on random time series segment extraction, rather than a forecasting model (for example, the Global Climate Models) is threefold: (a) Natural systems are inherently complex, nonlinear, and random. Forecasting based on observed time series is often more accurate and reliable because historical data samples have the same likelihood of recurring in the future as the original observations; (b) forecasting models for short time intervals-such as hourly or daily time series- are too complex to fully capture all the information contained in observed historical data. Stochastic processes, by extracting time series segments from historical records, preserve the full range of information, including extreme values that are difficult to simulate using traditional models; (c) model parameters can be biased or affected by trends and jumps in the observed data, reducing the reliability of such models^20^; In contrast, the stochastic process approach retains the integrity of the observed time series, making it less prone to errors introduced by data anomalies.

Stochastic forecasting utilizes historical record samples that are likely to exhibit similar statistical characteristics in the future. Through the application of Monte Carlo sample generation, a sufficiently large number of forecasted samples is generated, allowing for the creation of frequency distributions of different time-series metrics, such as mean and variance, and auto-covariation across different frequencies^20^. These measures are often challenging to calculate using alternative methods for generating stochastic time series.

Let $\:{\xi\:}_{T}\ge\:0$ denote the historical runoff time series for the time horizon of the whole dataset, T. $\:{t}_{0}$ stands for the time when historical data of the runoff begin. A set of an arbitrary sequence of times $\:{t}_{i}$ is selected between $\:{t}_{0}$ and *T*, *i* is the index for the selected time. One set of randomly generated runoff with n samples for a forecasting horizon $\:{T}_{H}$ can be expressed as $\:\left\{{\xi\:}_{{t}_{i}},\:{\xi\:}_{{t}_{i}+1},\:{\xi\:}_{{t}_{i}+2},\dots\:,\:{\xi\:}_{{t}_{i}+{T}_{H}-1}\right\}$, where index *i = 1*,* 2*,* 3*,*…*,* n*.

Based on the biennial periodicity in runoff, two classifications of runoff data can be extracted from the historical time series. One is the wet years’ data $\:{\xi\:}_{T}^{wet}$, and the other is the dry years’ data $\:{\xi\:}_{T}^{dry}$, which reflecting the biennial hydroclimate mode. For stochastic forecasting of a wet year, it can be expressed as $\:\left\{{\xi\:}_{{t}_{i}}^{wet},\:{\xi\:}_{{t}_{i}+1}^{wet},\:{\xi\:}_{{t}_{i}+2}^{wet},\dots\:{,\xi\:}_{{t}_{i}+{T}_{H}-1}^{wet}\:\right\}$, where index *i = 1*,* 2*,* 3*,*…*,* n*, and $\:{t}_{i}$ is selected only from wet years. A dry year forecasting of runoff is $\:\left\{{\xi\:}_{{t}_{i}}^{dry},\:{\xi\:}_{{t}_{i}+1}^{dry},\:{\xi\:}_{{t}_{i}+2}^{dry},\dots\:{,\xi\:}_{{t}_{i}+{T}_{H}-1}^{dry}\:\right\}$, where index *i = 1*,* 2*,* 3*,*…*,* n*, $\:{t}_{i}$ is selected only from dry years. Meanwhile, one question raised up, to what degree does the start month of the biennial periodicity impact the performance of the hydropower management? To investigate this question, this study considers the starting months of biennial periodicity for the forecasting samples used in stochastic forecasting. For example, one set of the forecasting samples for a wet year starting from January can be $\:\left\{{\xi\:}_{{t}_{i}}^{wet,Jan},\:{\xi\:}_{{t}_{i}+1}^{wet,Jan},\:{\xi\:}_{{t}_{i}+2}^{wet,Jan},\dots\:{,\xi\:}_{{t}_{i}+{T}_{H}-1}^{wet,Jan}\:\right\}$, where index *i = 1*,* 2*,* 3*,*…*,* n*, and $\:{t}_{i}$ is selected only from wet years January.

### Performance evaluation

The performance of the forecasting model is evaluated based on its accuracy, which is determined by the deviations between forecasting samples and the observed real data. Since real future data cannot be observed, a simulated “real” sample is generated to represent future observations. In this study, the real sample is represented by the mean value of runoff data selected from classifications corresponding to the scenarios.

The deviation between the forecasted samples and the simulated real data is quantified by the Nash-Sutcliffe efficiency coefficient (NSE), as shown in Eq. 1. NSE is widely used to measure the predictive skill of hydrological models, with values ranging from –∞ to 1, where NSE = 1 corresponds to a perfect match between the forecasts and the observed data^21–23^. An efficiency of 0 (NSE = 0) suggests that the model predictions are as accurate as the mean of the observed data, while an efficiency less than zero (NSE < 0) indicates that the residual variance is larger than the variance of the data.

However, NSE’s lower limit of negative infinity can complicate interpretation and presentation. To address this issue, the normalized Nash-Sutcliffe efficiency (NNSE) is used to rescale the NSE, as defined in Eq. 2. In this formulation, NNSE = 1 corresponds to NSE = 1, NNSE = 0.5 corresponds to NSE = 0, and NNSE = 0 represents NSE < -$\:\infty\:$. This rescaling simplifies the interpretation of the model’s performance.

1 $$\:NSE=1-\frac{{\sum\:}_{j=1}^{J-1}{({q}_{r,j}-{q}_{f,j})}^{2}}{{\sum\:}_{j=1}^{J-1}{({q}_{r,j}-{mean(q}_{f,j}))}^{2}}$$

2 $$\:NNSE=\frac{1}{2-NSE}$$

Where, *j* is the index of forecasting values, and *J* is the total number of forecasting values in one sample. $\:{q}_{r}$ stands for the simulated real runoff, and $\:{q}_{f}$ stands for the forecasted runoff.

Energy production efficiency ($\:\eta\:$) is defined as the potential production divided by the difference in potential runoff energy and potential storage energy, see Eq. 3. The potential production ($\:{E}_{pd}$) is the simulated downstream production, which indicates the estimated production generated at each station plus the potential production from all downstream stations along the water path towards the sea. The potential runoff energy ($\:{E}_{rd}$) and potential storage energy ($\:{E}_{sd}$) calculate for the entire downstream energy also, not for the individual station.

3 $$\:\eta\:=mean\left(\frac{{E}_{pd}}{{E}_{rd}-{E}_{sd}}\right)$$

### Hydropower operation model

The hydropower operation model developed by MATLAB R2022b for this study utilizes a step-linear optimisation approach, designed to optimise the planning and production of cascade hydropower plants and reservoirs. In practice, planning is equivalent to solving an optimisation problem, where the goal to achieve must adhere to certain constraints. The objective of the hydropower optimisation model is to maximize electricity production while maintaining the highest possible water levels in the reservoirs. The objective is mathematically expressed by the maximization of the objective function *F*, as defined in Eq. (4).

4 $$\:\underset{{t\:}\in\:{T}_{H}}{\text{max}}(F={\left({E}_{p}\right)}_{{T}_{H}}+{\left({E}_{w}\right)}_{{T}_{H}}-{\left({E}_{w}\right)}_{1})$$

5 $$\:{\left({E}_{p}\right)}_{{T}_{H}}={\sum\:}_{t=1}^{{t=T}_{H}}\rho\:g\eta\:hq\left(t\right){\Delta\:}t$$

6 $$\:{\left({E}_{w}\right)}_{{T}_{H}}={\sum\:}_{t=1}^{t={T}_{H}}\rho\:gAh{h}_{f}\left(t\right)$$

7 $$\:{\left({E}_{w}\right)}_{1}=\:\rho\:gAh{h}_{f}\left(1\right)$$

Where $\:{T}_{H}$(days) is the optimisation time horizon (equal to the forecasting horizon), $\:{\left({E}_{p}\right)}_{{T}_{H}}$(J) is the energy produced by hydropower for the entire optimisation time horizon $\:{T}_{H}$ (days), which can be calculated by Eq. (5). $\:{\left({E}_{w}\right)}_{{T}_{H}}$(J) is the potential water energy that is the energy stored in reservoirs for the period $\:{T}_{H}$ (days) and can be used to produce electricity in the future. $\:{\left({E}_{w}\right)}_{1}$ is the initial potential water energy. The potential water energy is calculated as a function of the downstream fall height $\:{h}_{f}$ (m), see Eq. (6), consisting of the water height from the station to sea level along with the downstream stations. $\:{\left({E}_{w}\right)}_{1}\:$is the initial potential water energy (Eq. (7)), the energy stored in reservoirs at the beginning of the hydropower optimal operation.

ρ (kg/m^3^) is the density of water; *g* (m/s^2^) is the acceleration due to gravity; and *η* is the constant generation efficiency of a hydropower plant; *h* (m) is the water level in a station; *q* (m^3^/s) is the turbine discharge; $\:{\Delta\:}t$ is the time period of energy production. A (m^2^) is the surface water area of each reservoir.

The constraints of the hydropower optimisation model comprise the conservation of water, which describes the dynamic water flows in the river basin between each hydropower station and the variables’ limitations, such as the turbine discharge and water head limitations. The constraints are recognized as Eq. (8)

8 $$\:{V}_{t}={V}_{t-1}+{q}_{up,t}{\Delta\:}t+{s}_{up,t}{\Delta\:}t+{q}_{r,t}{\Delta\:}t{-q}_{t}{\Delta\:}t-{s}_{t}{\Delta\:}t{V}_{t}=A{h}_{t}\underset{\_}{h}\le\:{h}_{t}\le\:\overline{h}0\le\:{q}_{t}\le\:\overline{\text{q}}\underset{\_}{s}\le\:{s}_{t}\le\:\overline{\text{s}}$$

Where $\:{V}_{t}$ is the volume of water in the reservoir at time index *t*. $\:{q}_{up,t}$ and $\:{s}_{up,t}$ are the turbine discharge and spillage discharge from the upstream station at time index *t*. The stations are connected by river channels and, therefore, have water flow from the discharge of the upstream stations. The water in upstream stations can be utilized to produce electricity several times when passing through several hydropower plants. $\:{q}_{t}$ and $\:{s}_{t}$ are the turbine and spillage discharges at the current station at time index *t*. $\:\underset{\_}{h},\:\overline{h}$ and $\:\underset{\_}{s},\:\overline{s}$ are the upper and lower boundaries of water level and spillage discharge. $\:\overline{q}$ is the upper boundary of turbine discharge. The travel time of water flow between stations is neglected, and the river channel is simplified as a rectangle.

### Model of assessing the hydroclimate periodic impacts

To assess the impact of hydroclimate periodic runoff on hydropower production and management, this study employs an assessment model designed to simulate the management efficiency of hydropower generation. This model accounts for the uncertainty in water availability forecasts and reflects the long-term periodicity observed in hydroclimatic time series. This assessment model, developed by Hao et al. 2023, consists of two main submodules: (a) an optimisation model, optimising hydropower operations by considering forecasted future runoff for *J* future states along a time horizon $\:{T}_{H}$, the water conservation of the river basin, and power production; and (b) a system update step, a submodule that updates the system after a shorter period $\:{t}_{u}$. The optimal production decisions are applied to the updating period$\:{t}_{u}$ for each $\:{T}_{H}$ horizon’s simulation based on the receding horizon method and the moving horizon of the several stochastic runoff forecasts along the time series covers a simulation period, $\:{T}_{sim}$ eventually. The updating includes the actual runoff and reservoir level, which aims to ensure that the effects of forecasting errors do not accumulate over time and thus represent the actual operational planning process over a more extended period. To provide a decision process that is statistically representative, the optimisation is carried out N times for each $\:{t}_{u}$ period with different stochastic forecasted runoff. Subsequently, the average values of these N optimal decisions of production and spillage discharges are applied as decided values for an updating time step, $\:{t}_{u}$.

**Fig. 1:**
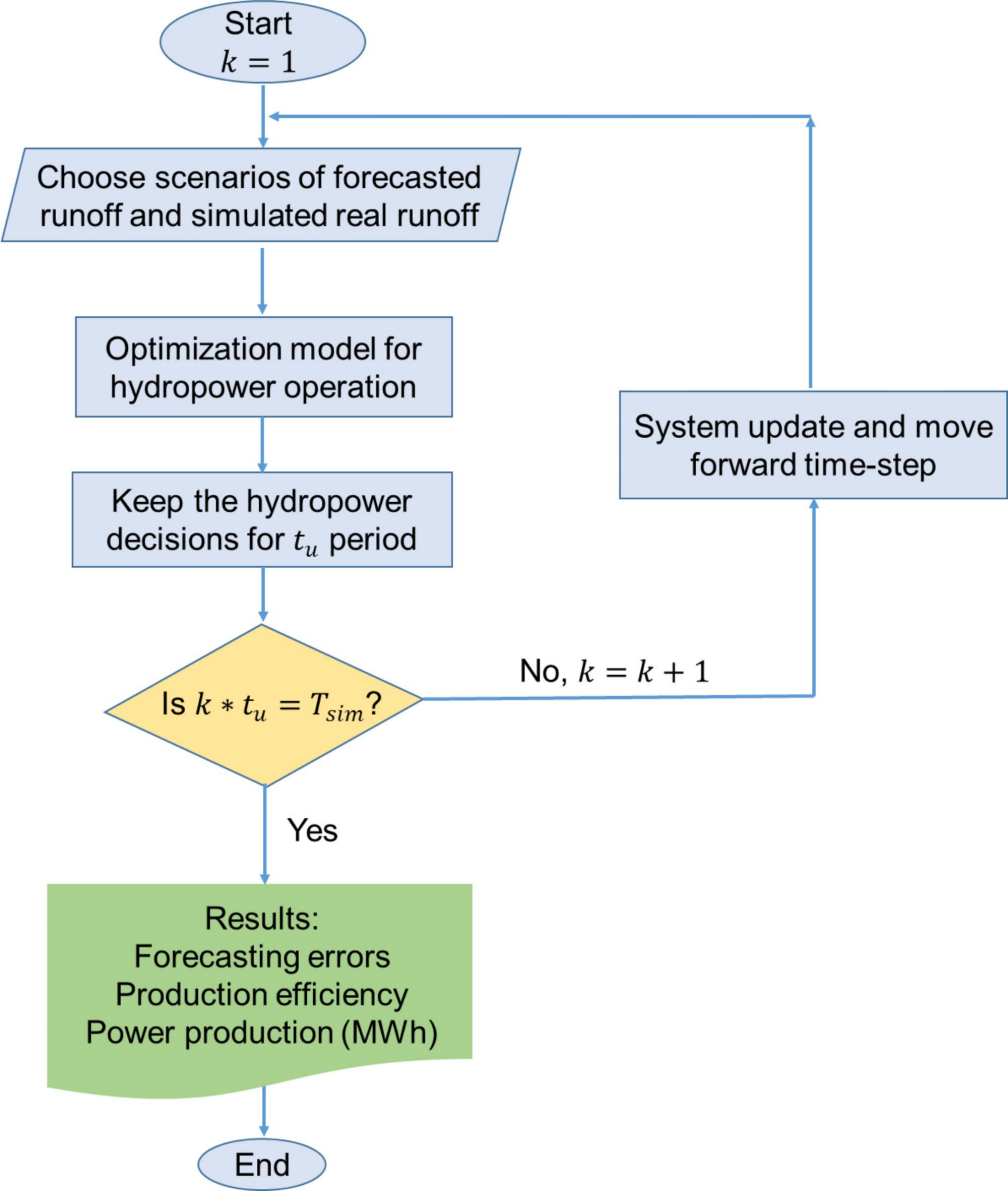
Flowchart of the assessment model.

The simplified model structure is illustrated in Fig. 1. The inputs are the forecasted runoff scenarios and the simulated real runoff scenarios for the time horizon $\:{T}_{H}$, generated using methods outlined in Sect. 2.1. Monte Carlo simulation is applied to runoff forecasting for the simulation period, $\:\:{T}_{sim}$, while the simulated real runoff is assumed to be a fixed time-series of runoff for each scenario. The outputs are the forecasting errors of the runoff, which is the deviation between forecasted samples and real samples of runoff, the production efficiency from the simulation of the hydropower optimal operation model, and the power production. The model parameters can be found in the supplementary information Table S1.

### Monte Carlo simulation

Monte Carlo (MC) simulation allows to characterise a distribution without needing to know its mathematical properties, by randomly sampling values from the distribution^24,25^. In this study, the impact of climate periodicity on hydropower management has been assessed through stochastic forecasting. A single random forecast is untenable to capture the whole picture of the impact. Hence, MC simulation is employed to explore a range of possible outcomes.

While MC simulation is advantageous for its ease of implementation with complex models, it requires substantial computational resources to yield reliable results^24,26^. To enhance the efficiency of the MC simulation of the assessment model, we conducted tests to determine the optimal number of MC runs. Specifically, we tested the Control-Control scenario with January as the start month, using a three-month simulation period, and varied the number of MC runs from 20 to 300. See Sect. 2.6 for detailed information. The variance, standard deviation and mean of the production efficiency are summarized in Table 1. The data indicate that the variance, standard deviation, and mean values stabilize with 100 MC simulations. Therefore, 100 MC runs are deemed optimal for balancing result reliability and computational efficiency. Consequently, all subsequent MC simulations in this study are conducted 100 times for each forecasting scenario.

**Table 1:** Statistic indicator for MC runs.

Number of MC	Variance	Standard deviation	Mean
20	0.0994186	0.3153	0.1569
40	0.1493877	0.3865	0.3140
60	0.1495559	0.3867	0.4713
80	0.0996968	0.3157	0.6283
100	0.0000089	0.0030	0.7854
120	0.0000084	0.0029	0.7854
140	0.0000087	0.0030	0.7854
160	0.0000082	0.0029	0.7854
180	0.0000088	0.0030	0.7854
200	0.0000089	0.0030	0.7855
250	0.0000097	0.0031	0.7854
300	0.0000097	0.0031	0.7854

### Hydroclimate biennial scenarios

The hydroclimate scenarios with biennial periodicity implemented in this study are based on the hydrologic stochastic forecasting method described in Sect. 2.1. This approach aims to separate the biennial periodicity in the runoff on dry and wet years. The forecasted runoff deviates statistically from the applied real runoff scenario. It has been observed that the odd-years are generally wetter than the average year, while even-years are generally drier for the Dalälven River Basin^5^. The historical records of runoff, therefore, can be divided into three classifications according to the biennial periodicity: Classification 1 contains the wet-year data; Classification 2 contains the dry-year data; Classification 3 is a control group data from, encompassing all years irrespective of whether they are odd or even.

The seven scenarios shown in Table 2 are developed based on these three classifications.

**Table 2:** Seven hydroclimate scenarios with biennial periodicity.

Scenarios	Descriptions
Wet-WetControl	Forecasting is from data classification 1, and simulated real runoff is the mean runoff of data classification 1
Dry-WetControl	Forecasting is from data classification 2, and simulated real runoff is the mean runoff of data classification 1
Dry-DryControl	Forecasting is from data classification 2, and simulated real runoff is the mean runoff of data classification 2
Wet-DryControl	Forecasting is from data classification 1, and simulated real runoff is the mean runoff of data classification 2
Wet-Control	Forecasting is from data classification 1, and simulated real runoff is the mean runoff of data classification 3
Dry-Control	Forecasting is from data classification 2, and simulated real runoff is the mean runoff of data classification 3
Control-Control	Both forecasting and simulated real runoff is from the data classification 3

## Case area and data

**Fig. 2:**
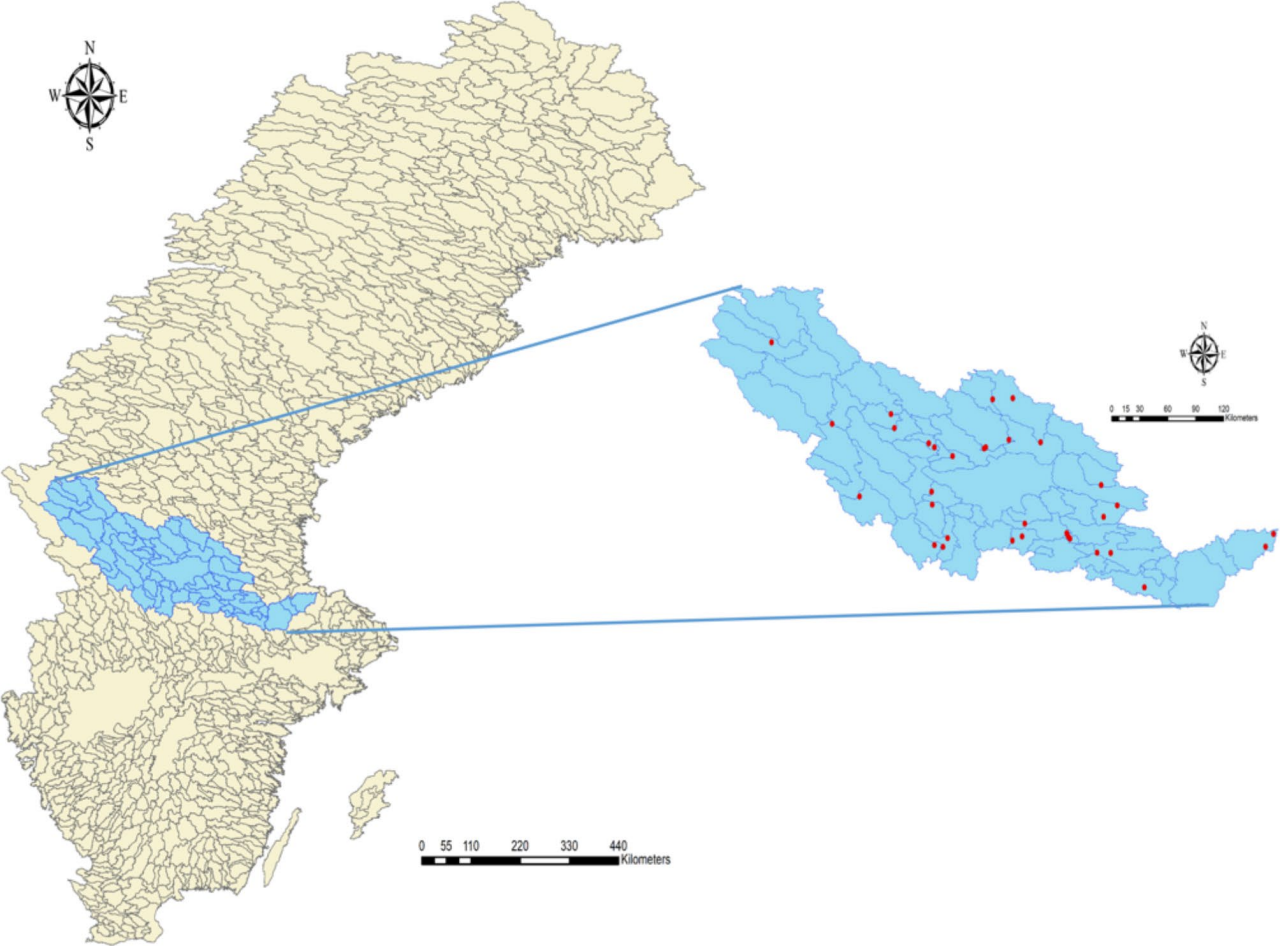
Map of Dalälven River Basin, created by ArcGIS 10.8.1 (https://www.esri.com/en-us/arcgis/products/arcgis-desktop/overview).

This study analyses data from the Dalälven River Basin, located in central Sweden. The basin features a main river channel that extends 541 km from the mountains of Dalarna to the Baltic Sea (Fig. 2). This study investigates 36 hydropower plants and 13 reservoirs in the basin. It is important to mention that not all reservoirs and hydropower plants in the Dalälven River Basin are included in this study; the hydropower plants and reservoirs used in this study are listed in the supplement information Table S2. The hydropower production model, described in Section. 2.3, is based on data from the Dalälven River Basin stations, including information on reservoir area, river channel distribution, water level limitations, and discharge constraints. The total hydropower production in the Dalälven River is approximately 4 TWh/year, and the installed hydropower capacity is 970 MW. The largest lake in this catchment area is Lake Siljan, which has a surface area of 354 km^2^, making it the seventh largest lake in Sweden.

The historical runoff data used in the stochastic forecasting model is collected from SMHI, the Swedish Meteorological and Hydrological Institute, and ranges from 1961 to 2011, spanning over half a century (51 years).

## Results and discussions

### Periodicity analysis

**Fig. 3:**
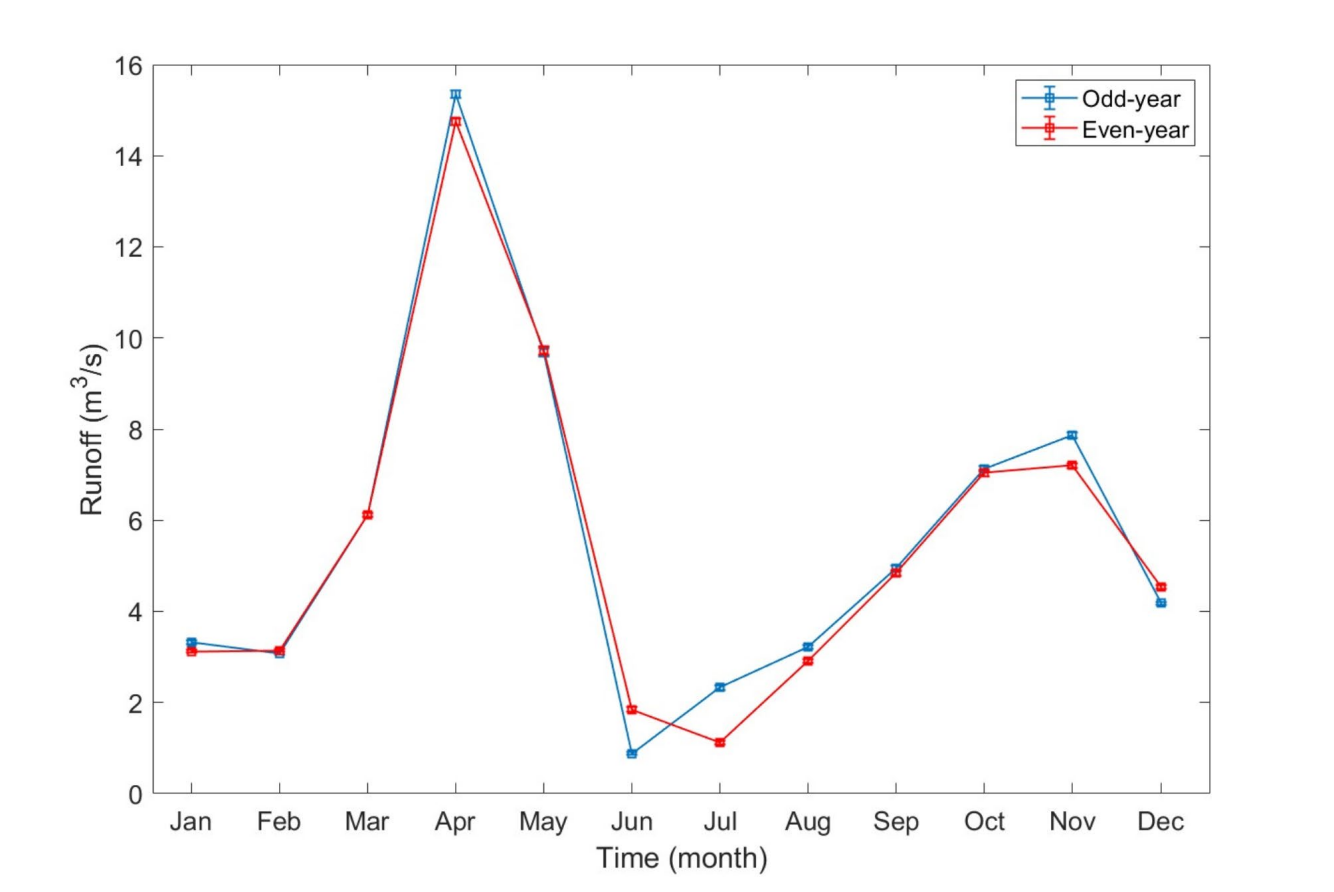
Monthly average daily mean runoff distribution for odd-year and even-year.

Biennial periodicity in hydrology and hydroclimate has been observed in numerous studies^8–10,27,28^. However, its characteristics, such as significance, duration and distinction between the wet and dry periods, vary depending on geographical and climatic factors. Hao et al. 2023 ^5^has shown the biennial periodicity in river discharge in the Dalälven River Basin by analysing different start months of the year and estimating the mean runoff for one year. This study evaluates monthly mean runoff data from odd and even years spanning 1961–2011, as illustrated in Fig. 3. It shows that the odd years are generally wetter, while even years are generally drier, with this effect being most pronounced during the peak flood months in April and November, as well as during low discharge period in summer. Notably, the wet and dry periods shift during drought months, such as June and July.

Runoff patterns have been demonstrated to be influenced by biennial periodicity linked to wet and given these observed runoff patterns, which are influenced by biennial periodicity, we hypothesize that cascade hydropower operation and production are similarly affected. This raises key questions about the extent to which biennial runoff periodicity impacts hydropower production and its effect on the accuracy of runoff forecasting. The following sections will address these inquiries in detail.

### Periodicity impact on hydropower production efficiency

To evaluate the impact of runoff periodicity on hydropower production, we evaluated the performance of hydropower operations, including energy production and production efficiency, under various scenarios: Wet-Control, Dry-Control, Wet-WetControl, Dry-WetControl, Dry-DryControl, and Wet-DryControl, as detailed in Sect. 2.6.

Each scenario is analysed over two simulation periods, $\:{T}_{sim}\:$, 90-day and 180-day, to assess the long-term effects on hydropower production.

The assessment model described in Sect. 2.4 employs runoff predictions that account for biennial periodicity to simulate hydropower production. Each scenario incorporates two sets of runoff data: forecasted future runoff and simulated real runoff. For the Wet-Control and Dry-Control scenarios, future runoff predictions are based on distinct classifications, while the simulated real runoff utilizes time-series data from the control classification. This comparison allows us to determine the impact of wet versus dry runoff forecasts on hydropower production and efficiency.

**Fig. 4:**
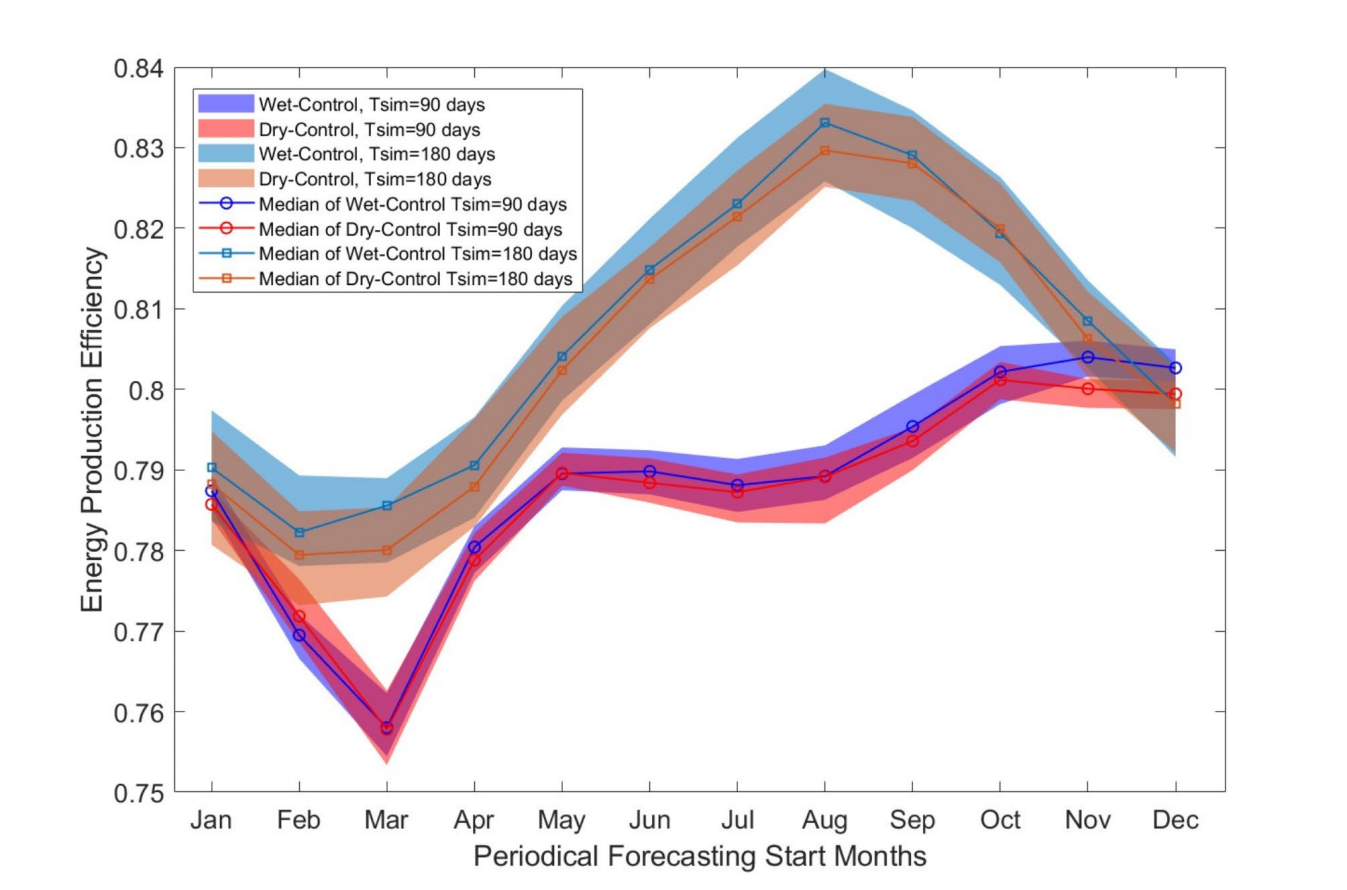
Range plot for production efficiency changing with operation planning start months for the length of 90-and 180-day simulation under the scenarios of Wet- and Dry-Control.

Figure 4 shows how production efficiency varies with different forecasts across twelve start months (January to December) and two simulation horizons ( $\:{T}_{sim}$=90 and 180 days). The figure shows clear deviations between wet-year and dry-year forecasts compared to the same control reality in both simulation periods. The blue area represents the wet-year forecasting, and the red area represents the dry-year forecasting.

In the context of a 90-day hydropower operation planning period, the biennial periodicity characterised by wet and dry patterns primarily affects production efficiency from May onwards. This influence extends throughout the hydropower operation phase from May to the following April. Minor variations in production efficiency are observed when operation planning begins between March and May. The median production efficiency shows the most substantial discrepancy of 0.4% variance (calculated as the difference between 0.804 and 0.800), between the Wet-Control and Dry-Control scenarios during the November to January planning period. For the 180-day simulation period, biennial periodicity influences efficiency throughout the year. Notably, from January to August, November, and December (the planning start month), the median production efficiency varies distinctly between wet-year and dry-year forecasts. It means that hydropower production is notably and consistently influenced by wet and dry years throughout the year, with relatively minor effects observed during the operation period starting in September and October.

**Fig. 5:**
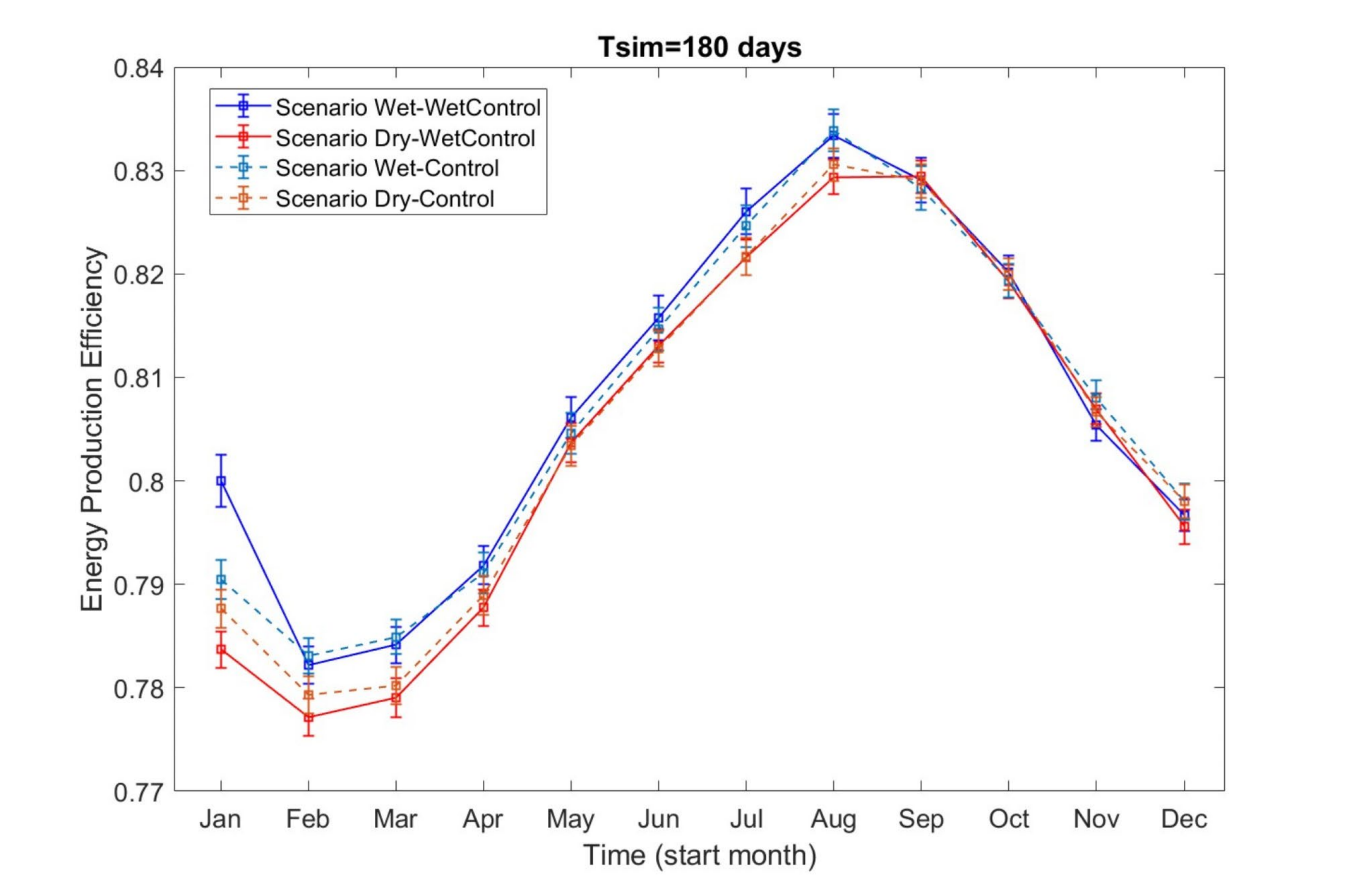
Production efficiency and its 95% confidence interval (assuming a normal distribution) vary with different operation planning start months for Wet-WetControl, Dry-WetControl, Wet-Control, and Dry-Control scenarios.

As shown in Fig. 5, using wet-year runoff forecasts for hydropower operations in wet years leads to a significant enhancement in production efficiency. The mean production efficiency increases by 1.63% (calculated as the difference between 0.8 and 0.7837), when compared to using dry-year forecasts in a wet year. The highest production efficiency is observed when employing wet-year forecasts in wet years (blue line), while the lowest efficiency occurs when using dry-year forecasts in wet years (red line). Meanwhile, the production efficiency of Wet-Control and Dry-Control scenarios falls between that of Wet-WetControl and Dry-WetControl scenarios. Notable deviations among these four scenarios are primarily observed when operation planning starts between January and August, affecting the hydropower operations throughout the year.

The peak period for production efficiency extends from August through February of the following year, while the lowest efficiency is observed from February to July.

For a 180 days hydropower operation during the winter months from September to December, the differences in production efficiency among the four scenarios are relatively small. This may be due to slow-flowing rivers and the accumulation of ice and snow during the winter, leading to minimal runoff variations across forecasting scenarios and, consequently, minor differences in hydropower production efficiency.

Additionally, wet-year forecasts consistently yield better production outcomes, irrespective of whether the year is a wet or control year. This is evident from the majority of the data, with the blue and blue dashed line consistently above the red and red dashed line.

**Fig. 6:**
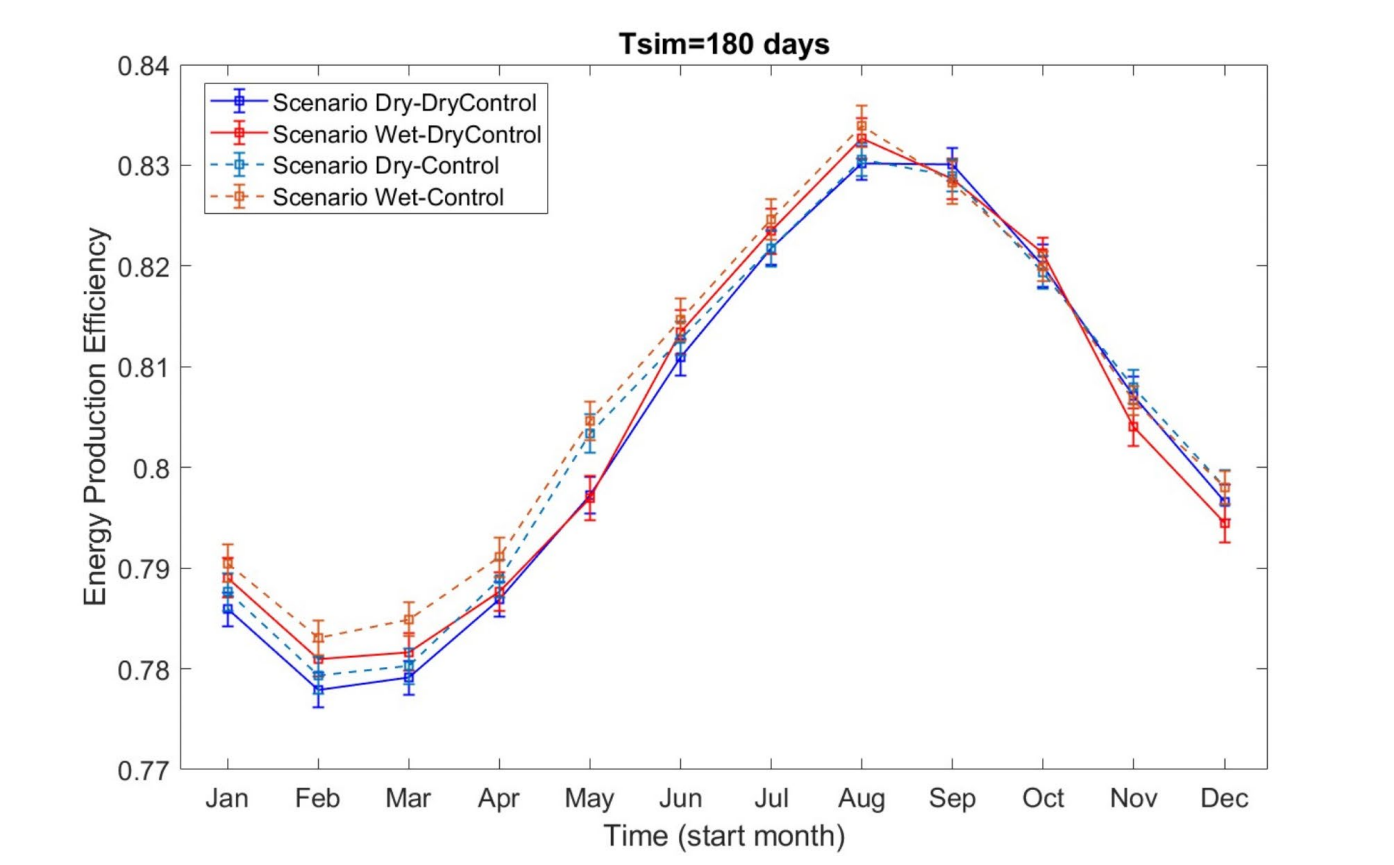
Production efficiency and its 95% confidence interval (assuming a normal distribution) vary with different operation planning start months for Dry-DryControl, Wet-DryControl, Dry-Control, and Wet-Control scenarios.

A similar analysis was conducted for the Dry-DryControl, Wet-DryControl, Dry-Control, and Wet-Control scenarios, as shown in Fig. 6. The findings show a consistent pattern: maximum hydropower production efficiency occurs between August and February of the following year, while relatively lower efficiency is observed from February to July. When comparing the Dry-DryControl and Wet-DryControl scenarios, it is evident that using wet-year runoff forecasts for hydropower operation in dry years (red line) results in a relatively higher production efficiency than using dry-year runoff forecasts (blue line). This suggests that misaligned dry-year runoff forecasting (when using wet-year forecasts to predict a dry year) may artificially inflate the production efficiency, particularly during the first half of the year. This effect is most pronounced when the 180-day simulation begins between January and March, with a maximum increase of 0.31%.

### Periodicity impact on hydropower production

**Fig. 7:**
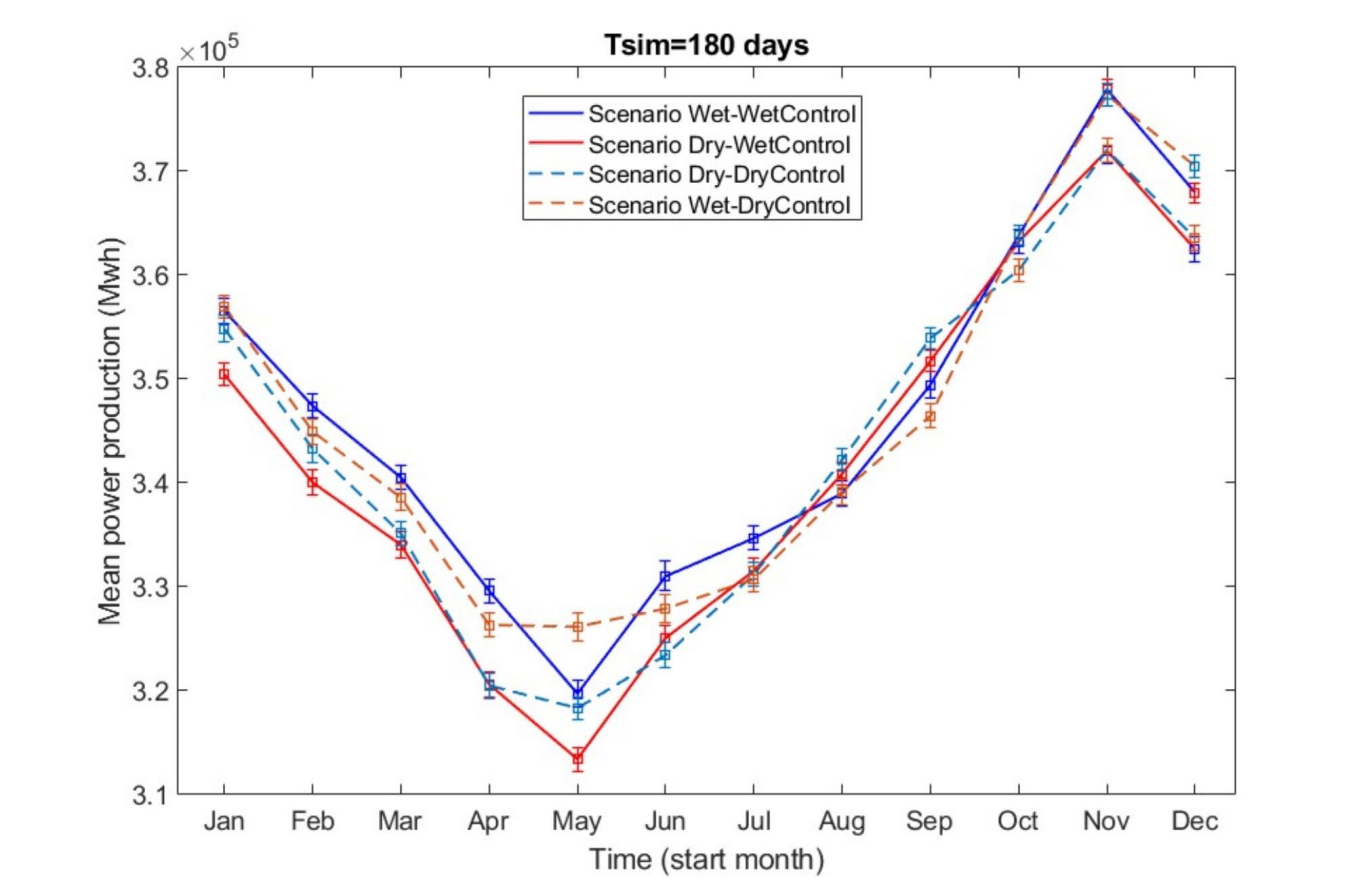
The mean power production and its 95% confidence interval (assuming a normal distribution) change with the operation planning start months for a 180-day simulation period.

Hydropower production exhibits a temporal pattern over a six-month simulation period, characterised by an initial decline, followed by a subsequent rise, and then another decline, as shown in Fig. 7. The minimum power generation occurs in hydropower simulations starting in May (range from 3.134*10^5^ to 3.262*10^5^ according to different scenarios), while the peak is observed in simulations beginning in November (range from 3.72*10^5^ to 3.779*10^5^). The most substantial impact on hydropower generation arises from misaligned wet-year runoff forecasts, where dry-year runoff is used to predict a wet year. This effect is particularly significant in simulations commencing from January through July, as well as in those starting in November and December over a six-month operational period. The most notable deviation between the Wet-WetControl and Dry-WetControl scenarios is seen during hydropower operations from April to September, wherein the application of a dry-year forecast in a wet year yields a decrease of 9104 MWh (calculated as the difference between 329611 MWh and 320507 MWh) in power production compared with the application of a wet-year forecast in a wet year. In contrast, the impact of misaligned dry-year runoff forecasting is less pronounced. As with the Wet-WetControl and Dry-WetControl scenarios, the distinction between the Dry-DryControl and Wet-DryControl scenarios in terms of hydropower production becomes particularly evident in simulations starting from January through July, as well as in November and December. The most significant difference occurs during hydropower generation from May to October, where using wet-year runoff predictions in dry years results in a spurious increase of 7832 MWh (calculated as the difference between 326163 MWh and 318331 MWh) in power production.

### Impact of periodicity on forecasting

The impact of periodicity on long-term runoff forecasting has been assessed by calculating the normalized Nash-Sutcliffe efficiency coefficient (NNSE). This metric is employed to gauge the variation between the forecasting samples and the simulated real data.

**Fig. 8:**
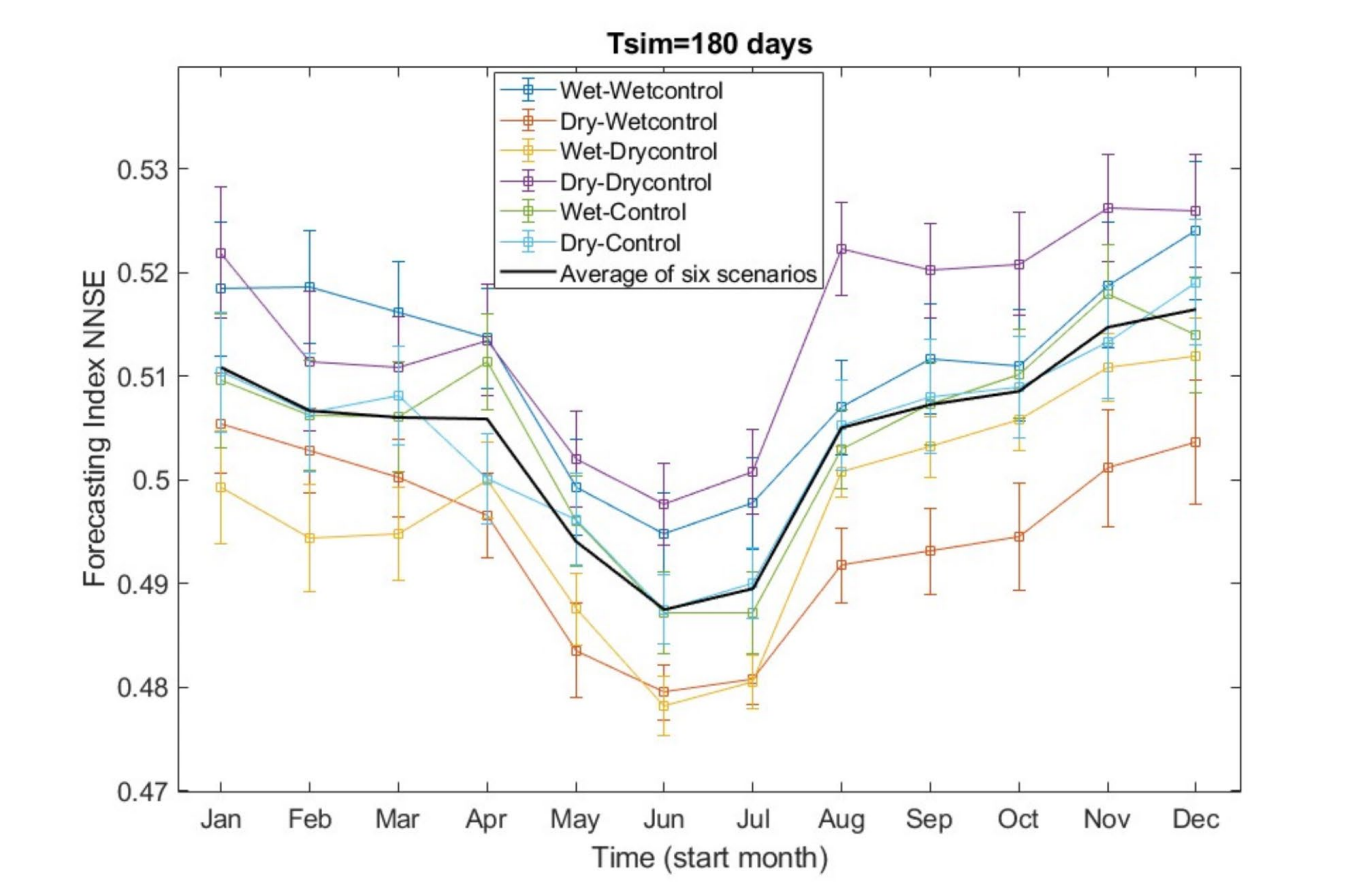
The NNSE forecasting index and its 95% confidence interval (assuming a normal distribution) varies with the operation planning period, represented by forecasting start months, for the 180 days simulation period.

Figure 8 presents the variation in NNSE over a half-year forecasting period, starting from different months. In terms of the NNSE index, higher NNSE values indicate better forecasting accuracy. The Scenarios analysed are Wet- and Dry-WetControl, Wet- and Dry-DryControl, and Wet- and Dry-Control.

As expected, employing wet-year forecasts in wet years and dry-year forecasts in dry years consistently shows a better performance in the forecasting index, across all scenarios. Dry-year forecasts in dry years produces the most accurate runoff forecasting, especially during the winter and spring seasons. Utilizing dry-year forecasts in a wet year yields the lowest forecasting index among all scenarios, with the second lowest forecasting index observed when employing wet-year forecasts in a dry year. This suggests that the misalignment of periodic runoff forecasting does indeed lead to a decrease in forecast accuracy, with the most significant decline reaching 3.05% (calculated as the difference between 0.5223 − 0.4918 for Dry-DryControl and Dry-WetControl scenarios in the period of August to January). It indicates this decline is particularly notable for the second half-year period from August to January, when dry-year forecasts are used for wet years, rather than employing wet-year forecasts.

The accuracy of the half-year runoff forecasting exhibits a trend of initially decreasing and then increasing with the progression of the prediction start month. The period from June to November shows the lowest forecast accuracy, with an average forecasting index NNSE of 0.4875 across all six scenarios. However, the forecasting from December to May achieves the maximum NNSE value, reaching 0.5164, in Fig. 8.

## Conclusions

The purpose of this study was to assess the impact of periodic forecasts on cascade hydropower operation and production, with a particular focus on long-term biennial runoff periodicity. Using the cascade hydropower system in the Dalälven River Basin as a case study, we investigated how different forecasting scenarios with biennial periodicity, for 90- and 180-day forecasts, affect power production efficiency, overall power production and forecasting accuracy. Based on 51 years of runoff data from the Dalälven River Basin, we employed hydrologic stochastic processes to generate synthetic runoff forecasts. These forecasts were classified by biennial periodicity. Seven forecasting scenarios were developed based on these classifications. The synthetic runoff forecasts from these seven scenarios were then used to drive an assessment model designed to simulate the management efficiency of hydropower generation, accounting for the uncertainty in water availability forecasts that reflect the long-term periodicity observed in hydroclimatic time series. The major findings from this study are:

1. Impact of biennial periodicity: the biennial periodicity of wet and dry years significantly affects production efficiency. Notable deviations between wet-year and dry-year forecasts were observed in both 90- and 180-day simulation periods. For the 90-day simulation, deviations are particularly pronounced from May onward and continue through the hydropower operation period from May to the following April, although production efficiency shows relatively minor variations when operation planning starts between March and May.
  For the 180-day simulation, biennial periodicity influences production efficiency throughout the year, with distinct variations between wet- and dry-year forecasts observed when simulations start from January to August, November, and December. The impact is less pronounced when operations begin in September and October.
2. Effect of misaligned runoff forecasts: misalignment between runoff forecasts and actual conditions has significant implications. Using dry-year runoff forecasts in wet years results in a substantial decrease in production efficiency and hydropower production, with an average reduction of 1.63% and a decrease of 9104 MWh, compared to using dry-year forecasts. Conversely, using wet year runoff forecasts in dry years artificially inflates production efficiency with 0.31% and spuriously increases 7832 MWh in power production. This underscores the importance of accurate runoff forecasting with respect to periodicity for optimising hydropower operation and production.
3. Forecasting accuracy: The normalized NSE index shows superior forecasting accuracy when wet-year forecasts are employed in wet years and dry-year forecasts in dry years. The relatively most accurate predictions are achieved by using dry-year forecasts in dry years, particularly during the winter and spring seasons. Conversely, employing dry-year forecasts in a wet year yields the lowest forecasting index.

This study demonstrates that integrating biennial periodicity into hydropower forecasting models significantly enhances the accuracy and efficiency of hydropower operations. By revealing the substantial impact of aligning forecasts with biennial runoff, the findings underscore the importance of incorporating long-term periodicity into management strategies. As climate variability continues to affect hydrological cycles globally, understanding and predicting these periodic patterns become crucial for optimising hydropower production. Accurate forecasting that reflects these periodicities not only improves production efficiency but also ensures more reliable management of hydropower resources. This study’s insights contribute to a broader understanding of how climate-driven periodicity influences energy production and highlight the need for advanced forecasting techniques in adapting to evolving climatic conditions. By addressing these periodic effects, hydropower systems can be better equipped to meet future energy demands sustainably. Future research could investigate other long-term periodicities that may impact hydropower production. Furthermore, improvements in forecasting techniques could be pursued to enhance the accuracy and reliability of predictions.

## Electronic supplementary material

Below is the link to the electronic supplementary material.

Supplementary Material 1

## Data Availability

The data, code, and models that support the conclusions of this research are available from the corresponding author upon request.
