# Supplementary material for: The impact of hydroclimate-driven periodic runoff on hydropower production and management
Source: Sci Rep. 2024 Oct 29;14:25967. doi: 10.1038/s41598-024-76461-3 (PMC11522470; doi:10.1038/s41598-024-76461-3)
Supplement: Supplementary file 1 — Supplementary Material 1 [file 41598_2024_76461_MOESM1_ESM.pdf]

The impact of hydroclimate-driven periodic runoff on hydropower production and management

Scientific Reports

Shuang Hao (shuangha@kth.se), Anders Wörman

Department of Sustainable Development, Environmental Science and Engineering (SEED)

KTH Royal Institute of Technology, 100 44 Stockholm, Sweden

Supplementary Information

The assessment model applied in this study aims to assess the impact of periodic runoff forecasting on hydropower production and management. It has been developed by Hao et al. 2023. In the assessment model, the parameters and their definitions are listed in Table S1. The reservoirs and hydropower plants in Dalälven River Basin that are included in this study are shown in Table S2

Table S1. Parameters of the assessment model

Parameter	Definition	Parameter value in the example application
T_H	Time horizon of optimisation: the duration of the forecasted time series placed into one optimisation procedure	T_H = 90 (days)
T_{sim}	Period of simulation: the maximum shift in time of the horizon in the receding horizon approach; $T_{sim} = K*t_u$	T_{sim} = 90 and 180(days)
t_u	Updating period: the time during which the decided turbine discharges are applied, whereafter the reservoir levels are updated and new decisions are taken; $t_u = T_{sim}/K$	t_u = 2 (days)
Δt	Numerical time step used to represent the watershed dynamics and to move between the states used in the optimisation	Δt = 0.5 (days)
j	j = 1:J. Index for the numerical time step for water dynamics; $J = T_H/\Delta t$.	J = 180
i	i = 1:M. Index for the reservoirs	M = 49
n	n = 1:N. Index for the repetition number of one updating period simulation with different stochastic runoff forecasts, used for making the average decision	N = 10

k	K = 1:K. Index for the simulation time step in order to progress over the simulation period T_{sim}. The number of updating time steps is $K = T_{sim}/t_u$.	K = 45

Table S2. The reservoirs and hydropower plants in Dalälven River Basin

Reservoirs	Hydropower Plants
Horrmundsjön	Horrmund
Öjesjön	Lima
Venjanssjön	Hummelfors(en)
Snesen	Eldforsen
Flaten	Johannisholm
Trängseltsjön	Gävunda
Vässinjärvi	Skifsforsen
Skattungen_Oresjön	Mockfjärd
Siljan	Lindbyn
Ljugaren	Båthuströmmen
Amungen	Trängslet
Balungen	Åsen
Runn	Väsa
	Blyberg
	Spjutmo
	Vässinkoski
	Noppikoski
	Furudal
	Skattungbyn
	Unnån
	Hansjö
	Gråda
	Forshuvud(forsen)
	Kvarnsveden
	Bullerforsen
	Domnarvet
	Dalstuga
	Tänger
	Borgärdet
	Sundborn
	Korsnäs
	Långhag
	Skedvi
	Avesta_Storfors
	Untra
	Lanforsen
